# Relationship between Sensory Processing and Quality of Life: A Systematic Review

**DOI:** 10.3390/jcm10173961

**Published:** 2021-08-31

**Authors:** Borja Costa-López, Rosario Ferrer-Cascales, Nicolás Ruiz-Robledillo, Natalia Albaladejo-Blázquez, Monika Baryła-Matejczuk

**Affiliations:** 1 Department of Health Psychology, University of Alicante, 03690 Alicante, Spain; borja.costa@ua.es (B.C.-L.); natalia.albaladejo@ua.es (N.A.-B.); 2 Institute of Psychology and Human Sciences, University of Economics and Innovation, 20-209 Lublin, Poland; monika.baryla@wsei.lublin.pl

**Keywords:** sensory processing, high sensitivity, quality of life, systematic review

## Abstract

Background: Sensory processing has been described as the ability to register, modulate, and organize sensory information to respond to environmental demands. Different theoretical approaches have studied the differential characteristics of sensory processing, such as Dunn’s model. From this framework, high sensitivity in sensory processing has been described as responses to stimuli from environment quite often due to a rapid activation of the central nervous system. It should be noted that the association between high sensitivity in sensory processing and health outcomes obtained in different studies are not homogeneous, so it is necessary to develop a review of this research in order to clarify the relationship between sensory processing and quality of life. Methods: We conducted a systematic review of the relevant studies using the PubMed, ScienceDirect, Scopus, and ProQuest databases to assess how sensory processing patterns are related to quality of life. Results: Fourteen studies concerning sensory processing and quality of life were included in the review. Some studies indicate negative, moderate, and significant correlations between these variables in which high sensitivity is related to a poor quality of life in the population studied. Conclusions: High sensitivity in sensory processing could have a negative impact on quality of life, thereby facilitating a fluctuation in well-being, daily functioning, and health.

## 1. Introduction

Sensory processing has been referred to as the ability to analyze, modulate, and organize sensory incoming information to respond to environmental stimuli [1]. This term has been determined over years by different authors [2,3,4,5,6,7,8,9]. Different theoretical approaches support that sensing and perceiving environmental stimuli is a programmed and survival way of functioning in humans to reach adaptation to the context [3,4,10]. However, although humans are neurobiologically predisposed to environmental survival, differences have been found in the way in which individuals react to the environment, since some people seem to have more sensitive brains [9,10].

Likewise, sensory processing patterns have been also identified by the four-factor Dunn’s model [10]. In this model, Dunn [10] describes the relationship between people’s neurological thresholds and behavioral responses from self-regulation strategies. From this view, four sensory patterns have been determined. The first two of them are associated with low sensitivity:

(1) Low registration, which means humans present high neurological thresholds and passive self-regulation strategies. It is known that they tend to have an uninterested appearance and to be underreactive. Hence, sensory profile research studies have linked this pattern to low endurance for tasks and poor registration of environmental stimuli.
(2) Sensation seeking, which is represented by people with high neurological thresholds and active self-regulation strategies. This pattern is recognized for presenting motor disorganization and impulsivity.

The other two patterns of Dunn’s conceptualization are related to high sensitivity:

(3) Sensation avoiding, which features exposure limitations to environmental stimuli. Individuals pretend to avoid the activation of their thresholds. Data from research articles have associated it with emotional reactivity.
(4) Sensory sensitivity, which is characterized by discomfort and overwhelming sensations in individuals. These people have low neurological thresholds, so they tend to be overreactive.

According to this, low or high sensitivity in sensory processing could be associated with self-regulation strategies to cope with environmental information [10]. Moreover, this theory introduces the importance of neurological thresholds to understand sensory processing [11,12]. Thresholds are on a continuum, and when a person has a low sensory threshold, it means that person notices and responds to environmental stimuli more often due to the rapid activation of the system [10,11,12]. That person is considered to present high sensitivity in sensory processing [12]. In this way, people have particular ways of responding to sensory events in daily life, representing a bell curve continuum from low to high with moderate or intense responses to sensory experiences [11,13]. Therefore, a wide range of possible interpretations of behaviors emerges, representing individual differences [10]. In fact, differences in sensory processing may be associated with different personality and temperamental dimensions [2,3].

As indicated, it is usual that individuals present a variation in their sensory processing patterns; however, a higher degree of sensitivity in sensory processing might predispose health implications and the development of psychopathology [6,9,10,14,15,16,17]. In this regard, a low threshold of sensory processing is known as a factor that could impact negatively on well-being, life satisfaction, quality of life (QOL), and health-related quality of life (HRQOL) [2,3,6,8,9,17,18,19,20,21,22].

Thus, higher levels of sensitivity in sensory processing are related to poorer QOL in several domains, including physical, cognitive, emotional, and social areas [23]. It is especially observed that work and household performance are interrupted [22,24,25]. Physically, high sensitivity has demonstrated some implications such as difficulties and exhaustion in sensory signal integration [2,14]. Physical fatigue is also identified in people with hypersensitivity due to prolonged period with no rest and being involved in stimulating contexts [26]. As recent studies have indicated, sensory processing is also linked to cognitive processing. For instance, abilities such as decision-making could be affected due to changes in the neural stream while processing environmental inputs [26,27]. Moreover, high levels of sensitivity in sensory processing could be related to low compassion satisfaction and high cognitive fatigue (quality-of-life factors) due to deep cognitive processing, perfectionism, and mental rigidity [14,28,29]. They keep directing their attention from one stimulus to the next [10]. In the emotional area, people who are hypersensitive can be fearful and become easily upset or even negative and defiant because of their emotional self-regulation [10,30]. Research studies have referred to this as an “emotional outburst” since they feel uncomfortable meeting their neurological thresholds [10]. Therefore, hypersensitivity is also manifested in the social area such as social distraction, isolation, and lack of communication skills [31,32]. These individuals’ sensory sensitivity can also interfere with academic or occupation performance and leisure participation [30]. These behaviors could result in maladjusted social management to environmental stimuli [26].

However, low sensitivity in sensory processing could also have negative consequences. Hence, previous research has stated that lower sensitivity could come with a high sensory threshold, which implies that more environmental inputs are needed to register them [22,33,34]. In fact, hyposensitivity could be featured by failing to detect sensation and not actively seeking sensory inputs [10]. Hence, low-sensitivity people have been found to have lower emotional and mental health-related quality of life (HRQOL), since they appear to be easily exhausted and apathetic, and need highly salient stimuli to engage them [22,25]. In fact, low levels of sensitivity could be associated with the inhibition of sympathetic nervous system activity, resulting in a decrease in daily social participation [22,35,36].

Nevertheless, although the issue is relevant among authors in terms of health, well-being, and QOL, current research has left a void in understanding sensory processing, the mechanistic understanding of environmental influences, and its implications in health. Despite a few studies suggesting that sensory processing patterns may be associated with positive outcomes, more research is needed. Moreover, the lack of terminological homogeneity makes it necessary to review research related to sensory processing, allowing an increase in evidence that sensory processing patterns might influence individuals lives. Therefore, the objective of this study was to assess the association between sensory processing patterns and the quality of life and health-related quality of life of the population.

## 2. Materials and Methods

This study used a systematic review methodology based on the PRISMA statement [37]. This systematic review was registered in PROSPERO with the following ID CRD42021246385.

The quality of each primary study was assessed with different types of tools, depending on the design of the study. Thus, the Newcastle–Ottawa Scale (NOS) for cohort studies, the Appraisal tool for Cross-Sectional Studies (AXIS), and the Cochrane Collaboration Risk of Bias (ROB) tool for randomized trials were all applied [38,39]. The ROB tool for randomized trials includes 32 items covering 6 domains of bias, 3 domains and 16 items for cross-sectional studies, and 3 domains with 8 items for cohort studies. In randomized trials and cross-sectional studies, each item in the ROB tool is judged as having a high, low, or unclear ROB. A summary assessment is calculated based on the number of items assessed as having high, low, or unclear ROB.

### 2.1. Data Sources

The systematic search was carried out in the PubMed, ScienceDirect, Scopus, and ProQuest databases. Additional articles were identified by searching the references of other articles.

### 2.2. Search Strategy

The search strategy aimed to identify the published studies available in full text. A bulk search strategy was used, using both descriptors, keywords, and terms in the titles or abstracts, which were as follows: “sensory processing sensitivity,” “sensory-processing-sensitivity,” “high sensitivity,” “quality of life,” and “health-related quality of life,” joined by Boolean operators (AND, OR) as follows: (sensory processing sensitivity OR sensory-processing-sensitivity OR highly sensitive person OR high sensitivity) AND (quality of life OR health-related quality of life). The date of the last search was 27 January, 2021, and no time restrictions were made about the year of publication of the studies. Table 1 shows the search strategy used in the abovementioned databases.

### 2.3. Selection of Articles and Risk of Bias

Abstracts identified through the bibliographic search were independently evaluated by two authors to confirm the inclusion criteria. Two authors of this paper rated each included article independently, and discrepancies were resolved by agreement with the third author. Cohen’s Kappa statistic was calculated to assess interrater reliability for the Newcastle–Ottawa Scale (NOS), the Appraisal tool for Cross-Sectional Studies (AXIS), and the Cochrane Collaboration Risk of Bias (ROB) tool. The results showed an agreement between two raters between 0.6 and 0.85.

### 2.4. Inclusion and Exclusion Criteria

Inclusion criteria were (I) articles that were available in full text and written in English or Spanish, (II) articles in which the sensory processing was reported with a numerical value, and (III) articles in which the HRQOL or QOL was described through numerical values.

Exclusion criteria were (I) articles not related to the subject of the study, (II) articles that did not include results, (III) articles that were reviews or meta-analysis, and (IV) documents that were summaries of conferences.

### 2.5. Extracted Data

Data extraction was carried out by the lead author of the review, considering the design and objective of the study, sample size, number of groups in the study, participants’ mean age, country of origin, sensory processing values, and HRQOL or QOL values. 

### 2.6. Synthesis of Results

After completing the data extraction, the results were listed and compared regarding means, standard deviations, and correlations of sensory processing on QOL and HRQOL.

## 3. Results

**Figure 1:**
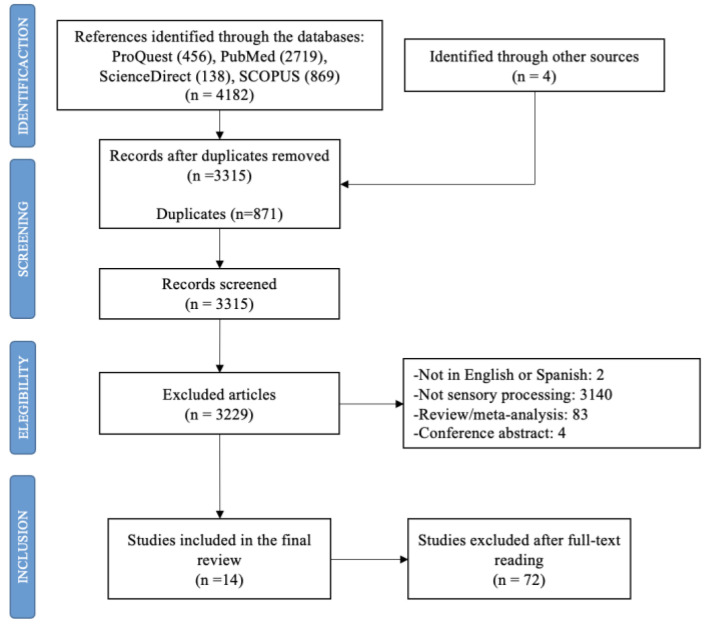
Flow diagram of study selection process.

In total, 4182 studies were identified. After the duplicates were removed (n = 871), the titles and summaries were read, and another 3229 articles were deleted according to the different exclusion criteria. Finally, 14 studies were included in this review (Figure 1).

### 3.1. Descriptive Data and Types of Studies

Table 2 shows the characteristics of the articles included. Of the participants, 57.91% were women (n = 1102) and 42.09% were men (n = 801), with a mean participant age of about 39.24 and a standard deviation of 14.11.

As for the country of origin, four of the articles were performed in the United States [25,40,41,42] and three studies were conducted in Israel [24,43,44]. Two articles were carried out in Italy [13,45], two in Australia [22,46], two in Korea [47,48], and one in Canada [49]. 

Table 2 also presents the design of the studies. It indicates that of the 14 studies included, 11 of them were cross-sectional [13,22,24,40,41,43,44,45,46,47,49]. Additionally, two studies were randomized studies [25,48] and one study was a cohort study [42]. 

**Table 2:** Description of the studies included in the review.

Authors, Year [Reference]	Country	Year	Age (Years)		Sample Size	Objective(s)	Design
			Mean (SD)	Range			
Kinnealey et al., 2011 [25]	USA	2011	SOR: 40.38 (±11.55) NSOR: 40.00 (±11.00)	18–60	Total: n = 28 Men: n = 7 Women: n = 21 SOR group: n = 14 NSOR group: n = 14	1. To explore the differences in social support and HRQOL between a group of adults with sensory modulation disorder (SOR) and a matched non-SOR (NSOR) group as well as the relationships between these variables. 2. To explore whether symptoms of anxiety and depression and indicators of HRQOL are significantly related to sensory response styles.	Randomized trial
Engel-Yeger et al., 2016 [13]	Italy	2016	Overall sample: 53.60 (±15.7) Unipolar group: 48.06 (±16.81) Bipolar group: 36.18 (±15.68)	16–85	Total: n = 267 Men: n = 93 Women: n = 174 Unipolar group: n = 157 Bipolar group: n = 110	1. To compare unipolar and bipolar patients with regard to their sensory processing patterns, coping strategies, and QOL. 2. To analyze the correlations between sensory processing patterns (dependent variable) and QOL (independent variable) in the total sample and among unipolar and bipolar patients while referring to coping strategies as a mediator variable. 3. To investigate the relative contribution of sociodemographic variables, groups (unipolar/bipolar), sensory processing patterns, and coping strategies in predicting QOL.	Cross-sectional
Pfeiffer et al., 2014 [41]	USA	2014	48.90 (±9.30)	-	Total: n = 94 Men: n = 48 Women: n = 46	1. To examine the relationship between different sensory processing patterns and community participation. 2. To inform the development of innovative rehabilitation interventions, including those resulting in more accommodating environments.	Cross-sectional
Engel-Yeger et al., 2017 [43]	Israel	2017	49.68 (±6.40)	33–55	Total: n = 115 Men: n = 50 Women: n = 65 Controlled diabetes group: n = 24 Uncontrolled diabetes group: n = 22 Healthy controlled group: n = 69	1. To profile sensory deficits examined in the ability to process sensory information from daily environment and discriminate between tactile stimuli among patients with controlled and un-controlled diabetes mellitus. 2. To examine the relationship between sensory deficits and patients’ HRQOL.	Cross-sectional
Colbeck, 2018 [49]	Canada	2018	-	>18	Total: n = 30 Men: n = 8 Women: n = 22	1. To describe how sensory-processing preferences and cognitive fatigue are related to variances in quality of life in people with multiple sclerosis (MS).	Cross-sectional
Genizi et al., 2019 [44]	Israel	2019	Migraine group: 10.06 (±1.53) Control group: 9.33 (±1.14)	7–12	Total: n = 134 Men: n = 59 Women: n = 75 Migraine group: n = 54 Control group: n = 80	1. To compare sensory processing abilities between children with migraine and healthy controls. 2. To compare the quality of life between children with migraine and healthy controls. 3. To examine the correlations between sensory processing, migraine characteristics, and quality of life among children with migraines. 4. To examine the contribution of headache-related disability and sensory processing to the prediction of quality of life among children with migraines.	Cross-sectional
Bar-Shalita et al., 2015 [24]	Israel	2015	27.30 (±3.77)	-	Total: n = 258 Men: n = 128 Women: n = 130	1. To explore in an ecological fashion the association between sensory responsiveness, pain perception, and QoL in subjects from the general population, with and without SMD. 2. To culturally adapt and initially test the Hebrew version of the PSQ.	Cross-sectional
Sinclair et al., 2019 [22]	Australia	2019	15.63 (±1.15)	13–18	Total: n = 70 Men: n = 7 Women: n = 63	1. To ascertain whether adolescents with persistent pain had atypical sensory modulation patterns. 2. To assess whether adolescents with persistent pain had atypical sensory modulation associated with reduced functioning and higher pain. 3. To evaluate whether pain catastrophizing adolescents mediate the relationship between sensory modulation and functional disability.	Cross-sectional
Crofton et al., 2020 [46]	Australia	2020	37.24 (±15.88)	18–76	Total: n = 117 Men: n = 86 Women: n = 31	1. To investigate associations between sensory variables and compression garment wear.	Cross-sectional
Stern et al., 2020 [42]	USA	2020	50.00 (±9.20)	23–65	Total: n = 94 Men: n = 15 Women: n = 79	1. To compare trait anxiety among persons with MS with different levels of sensory processing patterns. 2.To identify associations between sensory processing patterns, trait anxiety, and physical and mental HRQOL. 3. To explore the direct and indirect effects of sensory processing patterns on physical and mental HRQOL, considering trait anxiety as a potential mediator.	Cohort study
Serafini et al., 2015 [45]	Italy	2015	Overall sample: 48.31 (±11.47) Unipolar patient group: 43.19 (±13.03) Bipolar patient group: 35.12 (±14.66)	18–65	Total: n = 336 Men: n = 126 Women: n = 210 Unipolar patient group: n = 197 Bipolar patient group: n = 139	1. To compare unipolar/bipolar patients with regard to their sensory processing patterns, alexithymia, traumatic childhood experiences, and QOL. 2. To examine the correlations between sensory processing patterns and traumatic childhood experiences. 3. To investigate the relative contribution of diagnostic groups (unipolar/bipolar), sensory processing patterns, alexithymia, and traumatic childhood experiences in predicting QOL.	Cross-sectional
Eng et al., 2001 [40]	United States	2001	32.43	-	Total: n = 207 Men: n = 120 Women: n = 87	1. To examine potential differences on measures of the severity of social anxiety disorder, depression and functional impairment, and life satisfaction. 2. To corroborate the classification of attachment styles. 3. To explore the link between attachment styles and depressive symptoms within the sample of patients with social anxiety. 4. To examine the mediation of social anxiety in the relationship between adult attachment style and depressive symptoms in a clinical population.	Cross-sectional
Lee, 2012 [47]	Korea	2012	72.2 (±6.09)	>65	Total: n = 121 Men: n = 48 Women: n = 73	1. To assess the sensory processing ability of the normal elderly. 2. To express the importance of sensory integration in the elderly. 3. To understand the level of sensory defense. 4. To compare the correlation with quality of life.	Cross-sectional
Lee et al., 2016 [48]	Korea	2016	-	20–24	Total: n = 32 Men: n = 6 Women: n = 26 Sensory intervention group: n = 16 Control group: n = 16	1. To investigate the sensory processing abilities of university students. 2. To explore the influence of sensory processing on quality of life.	Randomized trial

Note. SOR = sensory overresponsive; NSOR = non-sensory overresponsive; SIG = sensory intervention group; CG = control group.

### 3.2. Sensory Processing Assessment Tools

Table 3 reports the type of instruments that were used to assess sensory processing in each of the studies included. Specifically, Table 3 shows 14 studies that applied instruments that follow Dunn’s sensory processing model [10]. These tools are the Adolescent/Adult Sensory Profile tool [12,13,22,41,42,43,45,46,47,48,49], the Short Sensory Profile [44,50], the Adult Sensory Questionnaire [25,51], the Sensory Responsiveness Questionnaire-Intensity Scale [24,52], and the Interpersonal Sensitivity Measure [40,53].

### 3.3. Quality of Life Assessment Tools

Table 3 also indicates the instruments used to assess QOL in each of the studies. For instance, among the studies, two articles reported applying different versions of the World Health Organization Quality of Life Questionnaire: One of them assessed QOL via the brief version [43,54], and the Korean version was also applied in one study [48,55]. Two studies used the Pediatric Quality of Life Inventory (PedsQL) [22,44,56]. Furthermore, the Short-Form-36 Health Survey (SF-36) [57] was used in two articles [24,25], and the 12-item Short-Form Health Survey version (SF-12) [58] was used in another two studies [13,45]. Additionally, in the rest of the studies, the RAND-36 [59], Assessment of Quality of Life-4D [60], Multiple Sclerosis Quality of Life-54 (MEQOL-54) [61], Lehman’s Quality of Life Interview [62], Quality-of-Life Inventory (QOLI) [63], and Elderly-people Quality of Life assessment tools [64] were applied [40,41,42,46,47,49].

### 3.4. Relationship between Sensory Processing and Quality of Life

As indicated in Table 3, in three studies, overall sample values were reported, in which moderate and negative correlations were found between sensory processing and quality of life [43,44,57]. Furthermore, in another six studies in which sensory processing was assessed by AASP [12], correlation values were noted through quality-of-life factors [13,22,42,43,46]. Thus, as presented in Table 3, almost every quality-of-life factor demonstrated negative correlations with sensory processing [13,22,42,49]. For the physical functioning factor, a positive correlation was found in two articles [13,49]. In fact, in the study by Crofton et al. [46], sensory processing correlated moderately and positively to quality-of-life factors such as help required, isolation, and anxiety. Table 3 also showed five studies that did not report correlations between sensory processing and QOL [24,40,41,45,48]. In addition, in three studies, samples with low levels of SPS indicated higher levels of quality of life or QOL factors [24,40,41] than those samples in which SPS levels were higher [45,48] (see Table 3).

**Table 3:** Sensory processing (Dunn’s model) and QOL assessment tools, means and SD of sensory processing and QOL, and correlations between these variables.

Authors, Year [Reference]		Sensory Processing Assessment Tool	QOL Assessment Tool	Sensory Processing Means (SD)	QOL Means (SD)	Correlations between Sensory Processing Patterns and QOL
Kinnealey et al., 2011 [25]		Adult Sensory Questionnaire (ASQ) [51]	Short-Form-36 Health Survey (SF-36) [57]	-	SOR = 18.81 (±3.88) NSOR = 21.80 (±2.03)	SOR sample: Physical functioning: r = −0.26 Bodily pain: r = −0.44* Vitality: r = −0.46 * Social functioning: r = −0.42 * Physical role: r = 0.19 Emotional role: r = −0.39 * Mental health: r = −0.35 General health: r = −0.40 *
Engel-Yeger et al., 2016a [13]		Adolescent/Adult Sensory Profile (AASP) [12]	12-item Short-Form Health Survey (SF-12) [58]	Unipolar patient group = 37.75 (±11.71) Bipolar patient group = 36.67 (±11.41)	Unipolar patient group = 56.69 (±35.58) Bipolar patient group = 55 (±35.41)	Physical functioning: r =− Bodily pain: r = −0.25 ** Vitality: r = −0.35 *** Social functioning: r = − Emotional role: r = −0.23 * Mental health: r = −0.24 ** Mental health composite: r = −0.30 ***
Engel-Yeger et al., 2017 [43]		Adolescent/Adult Sensory Profile (AASP) [12]	World Health Organization Quality of Life Questionnaire, brief version [54]	Controlled diabetes group = 39.45 (±8.82)	Physical QOL = 70.83 (±14.07) Psychological QOL = 78.33 (±12.48) Social QOL = 78.33 (±12.48) Environmental QOL = 77.08 (±20.31)	Overall sample: r = −0.477 **
				Uncontrolled diabetes group = 41.95 (±10.89)	Physical QOL = 57.49 (±19.07) Psychological QOL = 70.45 (±22.67) Social QOL = 61.74 (±26.18) Environmental QOL = 61.74 (±26.18)	
				Healthy controlled group = 34.57 (±6.51)	Physical QOL = 77.12 (±13.87) Psychological QOL = 74.78 (±12.02) Social QOL = 76.75 (±14.47) Environmental QOL = 70.83 (±13.28)	
Colbeck, 2018a [49]		Adolescent/Adult Sensory Profile (AASP) [12]	RAND-36 [59]	42.5 (±8.4)	-	General health: r = −0.65 *** Social functioning: r = −0.32 Pain: r = −0.14 Physical functioning: r = 0.06 Physical role: r = −0.19 Emotional role: r = −0.44 ** Fatigue: r = 0.08 Emotional well-being: r = −0.35
Genizi et al., 2019 [44]		Short Sensory Profile (SSP) [50]	Pediatric Quality of Life Inventory (PedsQL) [56]	Migraine group = 164.58 (±19.94) Control group = 174.11 (±9.35)	Total HRQOL: Migraine group = 8.26 (±12.13) Control group = 82.93 (±9.47)	Overall sample: Physical HRQOL: r = 0.45 *** Emotional HRQOL: r = 0.55 *** Social HRQOL: r = 0.31 School HRQOL: r = 0.44 *** Psychosocial HRQOL: r = 0.61 *** Total HRQOL: r = 0.63 ***
Sinclair et al., 2019 a [22]		Adolescent/Adult Sensory Profile (AASP) [12]	Pediatric Quality of Life Inventory (PedsQL) [56]	37.29 (±8.92)	Physical QOL = 44.38 (±21.43) Emotional QOL = 50.93 (±22.5) Social QOL = 68.93 (±23.74) School QOL = 45.07 (±21.62)	Physical QOL: r = −0.35 ** Emotional QOL: r = −0.41 *** Social QOL: r = −0.29 * School QOL: r = −0.32 **
Crofton et al., 2020a [46]		Adolescent/Adult Sensory Profile (AASP) [12]	Assessment of Quality of Life-4D [60]	-	-	QOL Help required: r = 0.148 QOL isolation: r = 0.361 ** QOL anxiety: r = 0.389 **
Pfeiffer et al., 2014 [41]		Adolescent/Adult Sensory Profile (AASP) [12]	Lehman’s Quality of Life Interview [62]	QOL scores: High sensitivity: 4.0 (±1.8) Low sensitivity: 4.7 (±1.4)		-
Bar-Shalita et al., 2015 [24]		Sensory Responsiveness Questionnaire-Intensity Scale (SRQ-IS) [52]	Short-Form-36 Health Survey, version 2 (SF-36) [57]	Bodily pain: Non-SMD = 80.2 (±21.20) SOR-SMD = 71.8 (±18.40) General health: Non-SMD = 79.5 (±17.97) SOR-SMD = 74.5 (±16.75) Vitality: Non-SMD = 54.7 (±17.65) SOR-SMD = 50.8 (±20.04) Social functioning: Non-SMD = 84.5 (±20.74) SOR-SMD = 80.5 (±19.81) Physical health–total: Non-SMD = 79.6 (±12.64) SOR-SMD = 74.4 (±13.51) Mental health–total: Non-SMD = 73.0 (±17.13) SOR-SMD = 67.3 (±16.30)		-
Stern et al., 2020a [42]		Adolescent/Adult Sensory Profile (AASP) [12]	Multiple Sclerosis Quality of Life-54 (MEQOL-54) [61]	-	-	MSQOL-54 physical: r = −0.43 *** MSQOL-54 mental: r = −0.52 ***
Serafini et al., 2015 [45]		Adolescent/Adult Sensory Profile (AASP) [12]	12-item Short-Form Health Survey (SF-12) [58]	Unipolar patient group = 37.55 (±11.58) Bipolar patient group: 36.09 (±11.38)	Body pain: Unipolar = 58.65 (±30.54) Bipolar = 56.75 (±29.27) General health: Unipolar = 64.14 (±24.01) Bipolar = 63.77 (±23.57) Vitality: Unipolar = 27.57 (±26.98) Bipolar = 33.13 (±30.31) Social functioning: Unipolar = 59.01 (±31.23) Bipolar = 53.19 (±36.15) Physical health–total: Unipolar = 48.72 (±11.95) Bipolar = 46.55 (±12.65) Mental health–total: Unipolar = 97.75 (±18.44) Bipolar = 96.16 (±25.04)	-
Eng et al., 2001 [40]		Interpersonal Sensitivity Measure (IPSM) [53]	Quality-of-Life Inventory (QOLI) [63]	Anxious attachment group = 108.69 (±11.86) Secure attachment group: 93.87 (±14.55)	Anxious attachment group = −0.56 (±1.38) Secure attachment group: 1.51 (±1.18)	-
Lee, 2012 [47]		Adolescent/Adult Sensory Profile (AASP) [12]	Elderly-people Quality of Life assessment tool [64]	-	-	r −0.30 **
Lee et al., 2016 [48]		Adolescent/Adult Sensory Profile (AASP) [12]	World Health Organization Quality of Life Questionnaire, Korean version [55]	Before sensory intervention SIG = 83.31 (±12.13) CG = 85.81 (±10.26)	After sensory intervention SIG = 98.69 (±11.67) CG = 84.81 (±14.56)	-

Note. SOR = sensory overresponsive; NSOR = non-sensory overresponsive; SMD = sensory modulation disorder; SIG = sensory intervention group; CG = control group. ^a^ Correlations between sensory processing and QOL as a general score are not reported, but they are reflected as correlations among SPS and some variable domains of QOL in the unipolar group. * *p* < 0.05. ** *p* < 0.01. *** *p* < 0.001.

## 4. Discussion

The present systematic review of 14 studies [13,22,24,25,40,41,42,43,44,45,46,47,48,49] indicates an association between sensory processing and quality of life. This study is the first systematic review investigation that collects the manifestation of the relationship between sensory processing and QOL from several studies.

As previously mentioned, these results present that sensory processing could be strongly correlated to quality of life and health-related quality of life. In fact, the existence of high levels of sensitivity of this feature could imply a decrease in quality of life and daily functioning based on the results obtained in the present review [13,22,25,42,43,46,47,49]. The studies analyzed show that high sensitivity may impact on health and well-being, since most of the studies demonstrated that physical, mental, emotional, and social areas are negatively influenced [13,22,24,25,40,41,42,43,46,47,49].

Especially in the physical area, some studies in the review stated that high levels of sensitivity in sensory processing might positively correlate to physical HRQOL (r = 0.45) [44]. Therefore, people with high sensitivity could be able to perceive subtle stimuli and delicate environmental stimuli, which can make them more alert to opportunities and rewards [14,19,65]. Nevertheless, other studies in this review found that the manifestation of high levels of sensitivity could negatively affect this physical functioning (from r = −26 to r = −43) [22,25,42]. Indeed, research studies have found that populations with hypersensitivity can suffer from physical fatigue, causing a decrease in their physical-related quality of life because of their large period of high stimulation with no rest [14]. In addition, although high sensitivity could increase the existence of physiological differences in stress-response systems and self-perceived stress, predisposing for physical symptoms and bodily sensations such as pain, just one of the included studies showed this positive correlation [17,66]. However, it seems that some of the studies in this review confirmed that low sensitivity to sensory processing might be related to less bodily pain [13,45,49].

Sensory processing is also manifested in the psychological and mental health area [2]. According to some included articles, high sensitivity could be negatively related to psychological QOL (from r = −0.24 to r = −0.52) [13,25,42,46]. In fact, recent studies have indicated that high sensitivity in sensory processing is featured as cognitive inflexibility and difficulties in decision-making due to deep processing and interpretation of environmental subtleties [14,29]. Consequently, this could make individuals with hypersensitivity prone to getting mental health and cognitive fatigue [8,34]. This could affect their executive functioning and their way of coping with tasks and daily situations [67,68]. Moreover, perfectionism, the need to control, and anxiety may be related to this issue [29], as indicated in Crofton et al. [46]. However, one of the studies in this review appeared to positively associate high levels of sensitivity in sensory processing with psychosocial HRQOL [44]. Previous studies explained that this ability might be connected to positive mental health outcomes [8,69]. In addition, the ability to search for similarities between current situations and previous ones was highlighted as a positive aspect [14]. In this way, people with high levels of sensitivity in sensory processing could more easily create associations and use comparisons and figurative schemes of coping [14].

Regarding the emotional area, despite part of the literature reporting that high sensitivity is related to deep emotional processes [2], some of the studies reviewed demonstrated that emotional quality of life could be negatively affected by sensory processing patterns (from r = −0.23 to r = −0.44) [13,22,25,49]. Hence, high sensitivity is related to intense reactions to images that evoke pleasant and unpleasant emotions [70]. In fact, when hypersensitive people get overwhelmed, they need to be careful in order to not get overly distressed and to develop appropriate coping and self-regulation strategies [71]. High levels of sensitivity in sensory processing might also be linked to socioemotional well-being, which can be manifested as a low self-esteem and shame, since they could feel socially judged by the environment and consequently misunderstood [14,17]. However, although this emotional part is “dark,” one of the included studies pointed out that people with high sensitivity in sensory processing patterns might be positively correlated with emotional HRQOL [44]. Besides, recent research has revealed that hypersensitive individuals could show emotionally positive aspects such as empathy towards others and a smart sense of humor [14,28]. Certainly, they can also more easily perceive emotions and emotional changes in others [16].

Additionally, in the social area, some articles demonstrated a negative relationship between high sensitivity in sensory processing features and social quality of life (from r = −0.27 to r = −0.42) [22,25,49]. As recent literature has described, people with high sensitivity tend to manifest with social distraction, avoidance of overstimulation, and a lack of communication skills to satisfy their social demands [31]. Moreover, Dunn’s model [10] has suggested that this population creates poor relationships due to low thresholds. They might present social reactivity and cope with social situations by manifesting fear, and in the end avoiding them [72,73]. Thus, high sensitivity in sensory processing may be also correlated with isolation, as one the studies included in this review showed [14,46]. Despite this, Genizi et al. [44] highlighted the positive association between high sensitivity in sensory processing and social HRQOL. Indeed, Serafini et al. [45] showed that the group with high levels of sensitivity presented better social functioning. That means that the population with hypersensitivity might be influenced by the environment in which they are involved [14]. Particularly, hypersensitive people who have grown up in adverse conditions are likely predisposed to suffer negative health consequences [31]. However, in supportive environments, individuals with high levels of sensitivity in sensory processing could improve their social competency and interpersonal interactions [72].

Finally, some articles did not present relationship values, so it is not clear whether sensory processing directly affected quality of life [24,40,41]. However, these studies showed that samples with high levels of sensitivity in sensory processing presented poorer quality of life.

### 4.1. Limitations

Among the possible limitations of this study, it should be mentioned that despite having carried out the search in four important databases, it is probable that other databases were not considered. In addition, although we used a wide range of descriptors and keywords to use a more precise strategy, there may be a specific keyword that was not controlled.

Another limitation to keep in mind is the heterogeneity of the sensory processing and QOL assessment tools applied. In fact, most standard deviations presented a wide range, which suggests that revising the occurrence of outliers in the data and the process of participant recruitment is necessary. Each questionnaire presents different structures and dimensions, and they were not created from a unique theoretical approach. Thus, the results from the articles compiled could be difficult to interpret.

This study evaluated the association between sensory processing and quality of life. However, just nine of the studies included presented this relationship with numerical values [13,22,25,42,43,44,47,49,56]. In this sense, it could be difficult to identify the relationship between sensory processing and QOL or HRQOL if studies do not report statistical indicators such as correlation values. Therefore, our results should be interpreted with caution.

### 4.2. Future Research 

Considering the aforementioned points, future investigation could continue research about sensory processing and its health implications. As indicated, statistical values should be thoroughly treated in order to improve the interpretation of sensory processing. Thus, future metanalytic studies should be considered to give a better comprehensive measure of the manifestation of sensory processing. This characteristic may have great influence on impacting the population’s quality of life and well-being [17]. Despite the “dark side” featured by negative consequences in humans, a “bright side” may exist that is not completely known yet due to its lack of evidence and could act as a protective factor [2,17]. Furthermore, sensory processing intervention programs could improve knowledge of it and favor self-regulation to prevent fatigue in hypersensitive populations. In fact, recent studies have mentioned the existence of some variables such as resilience, personal characteristics, and self-efficacy, as well as interventions based on mindfulness that could be trained to facilitate an increase in wellness [74,75].

## 5. Conclusions

Sensory processing is considered a feature that could be manifested in different life areas in the general population. People with hypersensitivity appear to negatively modulate health and quality of life, but recent studies have also demonstrated that it might show a “bright side.” This systematic review attempted to collect all the information about sensory processing features in order to clarify its role in people lives. Beyond considering it a disorder, high sensitivity in sensory processing has been determined to be part of personality that should be studied more in order to provide new light in research.

Heterogeneity in the results and statistical analysis also make the interpretation of the real implications of the variation in sensitivity of sensory processing difficult. For this reason, the conclusions of this study might be influenced by the data dispersion given the wide range of standard deviations. Therefore, more and more research studies should deal with this issue carefully to provide consistent results.

This study intended to present a theory background necessary to design a starting point. This could be useful to create new assessment tools and prevention and intervention programs to contribute to the knowledge and learning of this phenomenon and how to deal with it. Consequently, professionals and family could enrich their strategies to reach an improvement in their work and interpersonal relationships. In this sense, these actions might help hypersensitive people achieve an increase in health, well-being, and quality of life.

## Figures and Tables

**Table 1:** Database search strategy.

Search Strategy	
1.	(“sensory processing sensitivity” [All fields] OR “sensory-processing-sensitivity” [All fields] OR “highly sensitive person” [All fields] OR “high sensitivity” [Title/Abstract])
2.	(“quality of life” [All fields] OR “quality of life” [Title/Abstract] OR “health-related quality of life” [Title/Abstract] OR “health-related quality of life” [All fields])
3.	1. AND 2.

## Data Availability

Data sharing not applicable. No new data were created or analyzed in this study. Data sharing is not applicable to this article.

## References

[1] Humphry R. (2002). Young children’s occupations: Explicating the dynamics of developmental processes. Am. J. Occup. Ther..

[2] Ottoni G.L., Lorenzi T.M., Lara D.R. (2011). Association of temperament with subjective sleep patterns. J. Affect. Disord..

[3] Park C.I., An S.K., Kim H.W., Koh M.J., Namkoong K., Kang J.I., Kim S.J. (2015). Relationships between chronotypes and affective temperaments in healthy young adults. J. Affect. Disord..

[4] Shani-Adir A., Rozenman D., Kessel A., Engel-Yeger B. (2009). The relationship between sensory hypersensitivity and sleep quality of children with atopic dermatitis. Pediatr. Dermatol..

[5] Zald D.H. (2003). The human amygdala and the emotional evaluation of sensory stimuli. Brain Res. Rev..

[6] Rihmer Z., Akiskal K.K., Rihmer A., Akiskal H.S. (2010). Current research on affective temperaments. Curr. Opin. Psychiatry.

[7] Dunn W. (2002). The sensations of everyday life: Empirical, theoretical, and pragmatic considerations. Am. J. Occup. Ther..

[8] Pohl P.S., Dunn W., Brown C. (2003). The role of sensory processing in the everyday lives of older adults. Occup. Ther. J. Res..

[9] Engel-Yeger B., Gonda X., Walker M., Rihmer Z., Pompili M., Amore M., Serafini G. (2017). Sensory Hypersensitivity Predicts Reduced Sleeping Quality in Patients with Major Affective Disorders. J. Psychiatr. Pract..

[10] Dunn W. (1997). The impact of Sensory Processing Abilities on the Daily Lives of Young Children and Their Families: A Conceptual Model. Infants Young Child..

[11] Dunn W. (2007). Supporting Children to Participate Successfully in Everyday Life by Using Sensory Processing Knowledge. Infants Young Child..

[12] Brown C., Dunn W. (2002). The Adolescent/Adult Sensory Profile: User’s Manual.

[13] Engel-Yeger B., Gonda X., Muzio C., Rinosi G., Pompili M., Amore M., Serafini G. (2016). Sensory processing patterns, coping strategies, and quality of life among patients with unipolar and bipolar disorders. Rev. Bras. Psiquiatr..

[14] Pfeiffer B., Kinnealey M., Reed C., Herzberg G. (2005). Sensory modulation and affective disorders in children with Asperger syndrome. Am. J. Occup. Ther..

[15] Pertovaara A., Wei H. (2008). Dual influence of the striatum on neuropathic hypersensitivity. Pain.

[16] Fox M.A., Sanes J.R., Borza D.B., Eswarakumar V.P., Fässler R., Hudson B.G., John S.W., Ninomiya Y., Pedchenko V., Pfaff S.L. (2007). Distinct target-derived signals organize formation, maturation, and maintenance of motor nerve terminals. Cell.

[17] Brown N.B., Dunn W. (2010). Relationship between context and sensory processing in children with autism. Am. J. Occup. Ther..

[18] Dunn W. (2006). Sensory Profile Supplement: User’s Manual Blooming-Ton.

[19] Shochat T., Tzischinsky O., Engel-Yeger B. (2009). Sensory hypersensitivity as a contributing factor in the relation between sleep and behavioral disorders in normal school children. Behav. Sleep Med..

[20] Min C. (2017). Making Sense of Life Balance: A Coaching Intervention for Adults with Sensory Processing Challenges. Ph.D. Thesis.

[21] Rajaei S., Kalantari M., Azari Z.P., Tabatabaee S.M., Dunn W. (2020). Sensory Processing Patterns and Sleep Quality in Primary School Children. Iran. J. Child Neurol..

[22] Sinclair C., Meredith P., Strong J., Chalkiadis G.A. (2019). Sensory Modulation: An important piece of the disability puzzle for adolescents with persistent pain. Clin. J. Pain.

[23] Engel-Yeger B. (2012). Validating the Adolescent/Adult Sensory Profile and examining its ability to screen sensory processing difficulties among Israeli people. Br. J. Occup. Ther..

[24] Bar-Shalita T., Deutsch L., Honigman L., Weissman-Fogel I. (2015). Ecological aspects of pain in sensory modulation disorder. Dev. Disabil. Res. Rev..

[25] Kinnealey M., Koenig K.P., Smith S. (2011). Relationships between sensory modulation and social supports and health-related quality of life. Am. J. Occup. Ther..

[26] Engel-Yeger B., DeLuca J., Hake P., Goverover Y. (2021). The role of sensory processing difficulties, cognitive impairment, and disease severity in predicting functional behavior among patients with multiple sclerosis. Disabil. Rehabil..

[27] Alperin B.R., Tusch E.S., Mott K.K., Holcomb P.J., Daffner K.R. (2015). Investigating age-related changes in anterior and posterior neural activity throughout the information processing stream. Brain Cogn..

[28] Engel-Yeger B., Dunn W. (2011). The relationship between sensory processing difficulties and anxiety level in healthy adults. Br. J. Occup. Ther..

[29] Engel-Yeger B., Hus S., Rosenblum S. (2012). Age effects on sensory-processing abilities and their impact on handwriting. Can. J. Occup. Ther..

[30] Engel-Yeger B., Dunn W. (2011). Exploring the relationship between affect and sensory processing patterns in adults. Br. J. Occup. Ther..

[31] Engel-Yeger B., Palgy-Levin D., Lev-Wiesel R. (2015). Predicting fears of intimacy among individuals with post-traumatic stress symptoms by their sensory profile. Br. J. Occup. Ther..

[32] Stols D., van Heerden R., van Jaarsveld A., Nel R. (2013). Substance abusers’ anger behaviour and sensory pro- cessing patterns: An occupational therapy investigation. S. Afr. J. Occup. Ther..

[33] Brown C. (2002). What is the best environment for me? A sensory processing perspective. Ocuup, Ther. Ment. Health..

[34] Miller L.J., Anzalone M.E., Lane S.J., Cermak S.A., Osten E.T. (2007). Concept evolution in sensory integration: A proposed nosology for diagnosis. Am. J. Occup. Ther..

[35] Bar-Shalita T., Vatine J., Parush S. (2008). Sensory modulation disorder: A risk factor for participation in daily life activities. Dev. Med. Child Neurol..

[36] Chien C.W., Rodger S., Copley J., Branjerdporn G., Taggart C. (2016). Sensory processing and its relationship with children’s daily life participation. Phys. Occup. Ther. Pediatr..

[37] Moher D., Liberati A., Tetzlaff J., Altman D.G. (2010). Preferred reporting items for systematic reviews and meta-analyses: The PRISMA statement. Int. J. Surg..

[38] Downes M.J., Brennan M.L., Williams H.C., Dean R.S. (2016). Development of a critical appraisal tool to assess the quality of cross-sectional studies (AXIS). BMJ Open.

[39] Higgins J.P., Sterne J.A., Savović J., Page M.J., Hróbjartsson A., Boutron I., Reeves B., Eldridge S. (2016). A revised tool for assessing risk of bias in randomized trials. Cochrane Database Syst. Rev..

[40] Eng W., Heimberg R.G., Hart T.A., Schneier F.R., Liebowitz M.R. (2001). Attachment in individuals with social anxiety disorder: The relationship among adult attachment styles, social anxiety, and depression. Emotion.

[41] Pfeiffer B., Brusilovskiy E., Bauer J., Salzer M.S. (2014). Sensory Processing, Participation, and Recovery in Adults with Serious Mental Illnesses. Psychiatr. Rehabil. J..

[42] Stern B.Z., Strober L.B., Goverover Y. (2020). Relationship between sensory processing patterns, trait anxiety, and health-related quality of life in multiple sclerosis. J. Health Psychol..

[43] Engel-Yeger B., Darawsha S., Darawsha M. (2017). The relationship between health-related quality of life and sensory deficits among patients with diabetes mellitus. Disabil. Rehabil..

[44] Genizi J., Halevy A., Schertz M., Osman K., Assaf N., Segal I., Srugo I., Kessel A., Engel-Yeger B. (2019). Sensory Processing Difficulties Correlate with Disease Severity and Quality of Life among Children with Migraine. Front. Neurol..

[45] Serafini G., Gonda X., Pompili M., Rihmer Z., Amore M., Engel-Yeger B. (2016). The relationship between sensory processing patterns, alexithymia, traumatic childhood experiences, and quality of life among patients with unipolar and bipolar disorders. Child Abus. Negl..

[46] Crofton E., Meredith P.J., Gray P., Strong J. (2020). Compression garment wear and sensory variables after burn: A single-site study. Burns.

[47] Lee T.K. (2012). Correlations between Quality of Life and Sensory Processing Abilities in Older Adults. J. Korea Contents Assoc..

[48] Lee J.H., Lee T.Y., Kim Y.R. (2016). Quality of Life in Chungcheong area University Students according to their Sensory Processing Intervention. J. Korea Acad.-Industr. Coop. Soc..

[49] Colbeck M. (2018). Sensory processing, cognitive fatigue, and quality of life in multiple sclerosis. Can. J. Occup. Ther..

[50] Faul F., Erdfelder E., Lang A.G., Buchner A.G. (2007). A flexible statistical power analysis program for the social, behavioral, and biomedical sciences. Behav. Res. Methods.

[51] Kinnealey M., Oliver B., Wilbarger P. (1995). A phenomenological study of sensory defensiveness in adults. Am. J. Occup. Ther..

[52] Bar-Shalita T., Seltzer Z., Vatine J.J., Yochman A., Parush S. (2009). Development and psychometric properties of the Sensory Responsiveness Questionnaire (SRQ). Disabil. Rehabil..

[53] Boyce P., Parker G. (1989). Development of a scale to measure interpersonal sensitivity. Aust. N. Z. J. Psychiatry.

[54] World Health Organization (1996). World Health Organization Quality of Life Questionnaire, Brief Version (WHOQOL-BREF). http://www.who.int/mental_health/media/en/76.pdf.

[55] Min S.K., Kim K.I., Jung Y.C., Suh S.Y., Kim D.K. (2002). Development of the Korean versions of WHO Quality of Life scale and WHOQOL-BREF. Qual. Life Res..

[56] Varni J.W., Seid M., Kurtin P.S. (2001). PedsQL 4.0: Reliability and validity of the Pediatric Quality of Life Inventory version 4.0 generic core scales in healthy and patient populations. Med. Care.

[57] Ware J.E., Snow K.K., Kosinski M., Gandek B. (1993). SF–36 Health Survey Manual and Interpretation Guide.

[58] Ware J., Kosinski M., Keller S.D. (1996). A 12-Item Short-Form Health Survey: Construction of scales and preliminary tests of reliability and validity. Med. Care.

[59] Hays R.D., Morales L.S. (2001). The RAND-36 measure of health-related quality of life. Ann. Med..

[60] Hawthorne G., Richardson J., Osborne R. (1999). The Assessment of quality of life (AQOL) instrument: A psychometric measure of health-related quality of life. Qual. Life Res..

[61] Vickrey B., Hays R.D., Harooni R., Myers L.W., Ellison G.W. (1995). A health-related quality of life measure for multiple sclerosis. Qual. Life Res..

[62] Evensen J., Røssberg J.I., Barder H., Haahr U., ten Velden Hegelstad W., Joa I., Johannessen J.O., Larsen T.K., Melle I., Opjordsmoen S. (2012). Apathy in first episode psychosis patients: A ten year longitudinal follow-up study. Schizophr. Res..

[63] Frisch M.B. (1994). Quality of Life Inventory: Manual and Treatment Guide.

[64] Seong-jae C. (1986). Research on the development of the life satisfaction scale for the elderly people. Korean Cult. Res. Rev..

[65] Meyer B., Carver C.S. (2000). Negative childhood accounts, sensitivity, and pessimism: A study of avoidant personality disorder features in college students. J. Pers. Disord..

[66] Rappaport M.B., Corbally C. (2018). Evolution of religious capacity in the genus homo: Trait complexity in action through compassion. J. Relig. Sci..

[67] Engel-Yeger B., Rosenblum S. (2021). Executive dysfunctions mediate between altered sensory processing and daily activity performance in order adults. BMC Geriatr..

[68] Adams J.N., Feldman H.M., Huffman L.C., Loe I.M. (2015). Sensory processing in preterm preschoolers and its association with executive function. Early Hum. Dev..

[69] Boeke E.A., Moscarello J.M., LeDoux J.E., Phelps E.A., Hartley C.A. (2017). Active avoidance: Neural mechanisms and attenuation of pavlovian conditioned responding. J. Neurosci..

[70] Bailliard A.L., Whigham S.C. (2017). Linking neuroscience, function, and intervention: A scoping review of sensory processing and mental illness. Am. J. Occup. Ther..

[71] Aron E.N. (2002). The Highly Sensitive Child: Helping Our Children Thrive When the World Overwhelms Them.

[72] John T.S., Estes A., Begay K.K., Munson J., Reiter M.A., Dager S.R., Kleinhans N. (2021). Characterizing Social Functioning in School-Age Children with Sensory Processing Abnormalities. J. Autism Dev. Disord..

[73] Koenig K.P., Rudney S.G. (2010). Performance challenges for children and adolescents with difficulty processing and integrating sensory information: A systematic review. Am. J. Occup. Ther..

[74] De Vera M.I., Gabari M.I. (2020). Associated factors with resilience and burnout: A cross-sectional study in a teaching group in Spain. Aula Abierta.

[75] Hebert K.R. (2016). The association between sensory processing styles and mindfulness. Br. J. Occup. Ther..

